# A label-free fluorescent biosensor based on specific aptamer-templated silver nanoclusters for the detection of tetracycline

**DOI:** 10.1186/s12951-023-01785-7

**Published:** 2023-01-20

**Authors:** Si Yang, Chenxi Li, Hongyan Zhan, Rong Liu, Wenliang Chen, Xiaoli Wang, Kexin Xu

**Affiliations:** 1 State Key Laboratory of Precision Measuring Technology and Instruments, Tianjin University, Tianjin, 300072 People’s Republic of China; 2 School of Precision Instruments and Optoelectronics Engineering, Tianjin University, Tianjin, 300072 People’s Republic of China; 3 Department of Medical Imaging, Weifang Medical University, Weifang, 261053 Shandong People’s Republic of China

**Keywords:** Ag nanoclusters, Aptamer, Tetracycline, Biosensors, Fluorescence

## Abstract

Tetracycline (TET) is a broad-spectrum antibiotic commonly used in the treatment of animals. TET residues in food inevitably threaten human health. High-performance analytical techniques for TET detection are required in food quality assessment. The objective of this study was to establish a label-free fluorescent biosensor for TET detection using specific aptamer-templated silver nanoclusters (AgNCs). An aptamer with a high specific binding ability to TET was used to synthesize a novel DNA-templated AgNCs (DNA-AgNCs). When TET is present, the aptamer’s conformation switched from an antiparallel G-quadruplex to a hairpin structure, altering the connection between AgNCs and the aptamer. Following the transformation of AgNCs into large sized silver nanoparticles (AgNPs), a fluorescence decrease was detected. When used to detect TET in milk, the proposed biosensor displayed high sensitivity and selectivity, with a limit of detection of 11.46 ng/mL, a linear range of 20 ng/mL−10 g/mL, and good recoveries of 97.7–114.6% under optimized conditions. These results demonstrate that the proposed biosensor was successfully used to determine TET quantitatively in food samples, suggesting that our method provides an efficient and novel reference for detecting antibiotics in food while expanding the application of DNA-AgNCs in related fields.

## Introduction

Tetracycline (TET) is a popular antibiotic in the animal breeding industry due to its wide antimicrobial activity, low cost and relatively low toxicity [1]. The abuse of TET has led to antibiotic residues in various animal-derived foods, including meat [2], eggs [3], milk [4], and honey [5]. Accumulating with the food chain, TET inevitably threaten human health, resulting in negative health, such as allergic and toxic reactions, as well as kidney and liver damage [6]. TET residue limits of 100 µg/kg in milk have been established by the European Union (EU) and the Chinese Ministry of Agriculture [7]. Therefore, numerous efforts have been made to control TET in food to ensure food safety and safeguard consumer health.

High-performance liquid chromatography (HPLC) [8], capillary electrophoresis (CE) [9] and liquid chromatography–tandem mass spectrometry (LC–MS/MS) [10] have the benefits of high precision and sensitivity. However, these instrumental analysis methods are time consuming, laborious, and require skilled operators. Immunoassays provide a simple and economical alternative to instrumental methods, including gold immunochromatographic assay (GICA) [11], enzyme-linked immunosorbent assay (ELISA) [12], and fluorescent immunoassay (FIA) [13]. However, these immunoassay methods based on antigen–antibody interactions strongly depend on the quality of antibodies, which is laborious and batch-to-batch variation. Moreover, the application of these techniques in detection has been hampered by the strict detection conditions of antibodies, such as temperature, pH, and ionic strength.

Aptamers are single-stranded oligonucleotides that are selected from a pool of DNAs or RNAs sequences through systematic evolution of ligands by exponential enrichment (SELEX). Taking advantage of high binding affinity and specificity to target molecules with no restrictions on target size, chemical stability and high reproducibility, as well as wide chemical modification capability, aptamers could be capable of precise molecular recognition and can be conjugated with a variety of transduction technologies to create a wide range of diverse biosensors. The signal transduction strategies in these biosensors for the detection of TET mainly include electrochemical [14], colorimetric [15], and fluorescence [16] methods. Among these strategies, it is cumbersome and tedious to prepare electrodes for electrochemical methods, and sample color may affect colorimetric results. Due to the merits of easy modification of fluorophores by aptamers and the advantages of high sensitivity, fast analysis speed, simple instrumentation, and little damage to samples for fluorescence methods, aptamer-based fluorescent biosensors have attracted great attention [17, 18]. Correspondingly, fluorescent materials including organic dyes [19], quantum dots (QDs) [18], and upconversion nanoparticles (UCNPs) [15] have risen in popularity as biosensor materials. However, organic dyes have high toxicity and poor photostability, UCNPs have low luminous efficiency, and QDs, which area type of prelabeled signal source, are time-consuming, laborious, and costly. Therefore, there is a pressing need to develop non-toxic, low-cost and effective label-free fluorescent probes to compensate for these deficiencies.

As novel fluorescent nanomaterials, silver nanoclusters (AgNCs) have attracted tremendous attention, owing to high stability, biocompatibility, ultrasmall size, excellent dispersibility, facile synthesis, high quantum yield, excellent photostability and low toxicity [20]. However, AgNCs easily aggregate irreversibly in aqueous solution, resulting in an increase in size and loss of optical properties. Therefore, a variety of ligands have been utilized to stabilize AgNCs, such as polymers, proteins, short thiol compounds and DNA. The high affinity of Ag^+^ toward cytosine (C) and the abundant tertiary structures of DNA, such as G-quadruplexes [21] and i-motifs [22] have made DNA a superb template for AgNCs synthesis. In addition, the photoemissive wavelength of DNA-templated silver nanoclusters (DNA-AgNCs) can be tuned from visible to near-IR by varying the base sequence and length of the DNA template. Moreover, overcoming the shortcomings of fluorescent probes that need modification makes DNA-AgNCs excellent signal-reporter elements for biosensor construction.

There have been a few reports of label-free fluorescent biosensors based on DNA-AgNCs. For example, Yan et al. designed a hairpin structure fluorescent probe tagged at two terminals with DNA-AgNCs and guanine-rich sequences (GRS) based on the principle that guanine (G)—rich sequences could significantly boost the fluorescence of AgNCs for the determination of bleomycin (BLM) [23]. Miao’s group utilized two hairpin structures of signal probe and capture probe for the ratiometric detection of miRNA by switching the emission wavelength of DNA-AgNCs through the regulation of complementary DNA [24]. Jiang et al. designed two DNA sequences against carcinoembryonic antigen (CEA) and alpha-fetoprotein (AFP) containing aptamer sequences joined with two different AgNCs synthesis sequences and combined a novel fluorescence quenching material, polydopamine nanospheres (PDANs), to establish a sensing platform for multiple tumor marker detection [25].

Most biosensors constructed by DNA-AgNCs require auxiliary DNA to assist signal transduction, and fluorescent quenching materials to turn on fluorescence signals, making sensor assembly relatively complex. Moreover, in DNA-AgNCs probe, the recognition unit (the aptamer of analyte) and signal unit (DNA template for AgNCs) are integrated, which makes part of the DNA template affect the specific recognition ability of the aptamer, and the optical properties of AgNCs not be precisely controlled.

The objective of this paper was to utilize a label-free fluorescent biosensor with specific aptamer-templated AgNCs to detect TET. We established a unique approach for the synthesis of AgNCs based on the aptamer sequence of TET, which is rich in cytosine and capable of forming a G-quadruplex structure. The fluorescence emission wavelength of AgNCs (Apt76 - AgNCs) emitted at approximately 625 nm. The basic principle of the determination of TET is based on a detected reduction in the fluorescence of AgNCs caused by conformational change after binding of aptamer to TET accompanied by the size change of AgNCs. By integrating aptamers with AgNCs, a label-free fluorescence biosensor is created by combining the unique recognition properties of aptamers with the optical properties of AgNCs is assembled, which greatly simplifies the fabrication process of the sensing platform and shortens the detection time. In addition, the label-free sensing platform may also be adapted to detect additional targets that have aptamers with similar properties.

## Materials and methods

### Chemicals

Sangon Biotech. Co., Ltd. (Shanghai, China) developed the 76-mer ssDNA aptamers (Apt76) used in this study, which were purified using high-performance liquid chromatography (HPLC). Apt76 [26] had the sequence 5′-CGT ACG GAA TTC GCT AGC CCC CCG GCA GGC CAC GGC TTG GGT TGG TCC CAC TGC GCG TGG ATC CGA GCT CCA CGT G-3′. Tetracycline (TET), penicillin, ciprofloxacin, chloramphenicol, streptomycin, ofloxacin, kanamycin, silver nitrate (AgNO_3_) and sodium borohydride (NaBH_4_) were purchased from Sigma–Aldrich (St. Louis, USA). All of the compounds employed were analytical reagent grade and did not require additional purification.

### Apparatus

The excitation fluorescence spectra were measured with Spectra Max i3X (Molecular devices, USA) equipped with a fluorescent module at 25 °C. All fluorescence emission spectra were collected by scanning from 600 to 750 nm with a resolution of 1 nm at an excitation wavelength of 570 nm. UV-VIS spectrum was carried out with a spectrophotometer (UV-2550, Shimadzu, Japan). Circular dichroism spectra were obtained at 25 °C on a circular dichroism spectrometer (MOS-500, BioTools, USA) using a 1 mm optical path-length quartz cell, within the range of 210–350 nm at 1 nm intervals. Transmission electron microscopy (JEM-2100 F, JEOL, Japan) operated at 200 kV was used to measure the particle sizes of the produced DNA-AgNCs.

### Molecular docking study of Apt76 and TET

The molecular docking was simulated with AutoDock Tools to investigate the binding between TET and aptamer. The M-fold online web service predicted Apt76’s secondary structure, and TET and Apt76’s 3D structure data were downloaded from Pubchem and the 3dRNA/DNA Web Server, respectively. The conformations of TET were scored and ranked based on the the free energies of binding with Apt76. The docking result analysis and display were completed by PyMOL software.

### Preparation of Apt76-AgNCs

To begin, deionized water and buffer were added to 10 µL of 100 µM aptamer template of AgNCs, which was then heated at 95 °C for 10 min before being slowly cooled to 25 °C. Secondly, 2 mM AgNO_3_ solution was included in the aforementioned solution, followed by 1 min of vigorous shaking and 30 min of incubation in the dark at 4 °C. After that, freshly made 3 mM NaBH_4_ solution was added to the aforesaid solution, which was then shaken for 1 min. The combination was left in the dark for 4 h to generate AgNCs for future research.

### Optimization conditions for Apt76-AgNCs preparation

Buffer and the molar concentration of Apt76, Ag^+^ and NaBH_4_ significantly impact the fluorescence properties of Apt76-AgNCs. NH_4_Ac, PB and sodium citrate with pH 6.8, 7.2, 7.4, 7.6, or 8.0, were used to prepare DNA-AgNCs for comparison. Moreover, AgNCs synthesized at a molar ratio of Apt76:Ag^+^:NaBH_4_ of 1:8:3, 1:8:9, 1:12:3, 1:12:6, 1:12:12, 1:24:3, 1:24:12, or 1:24:24 were selected to obtain the optimal ratio by comparing the fluorescence intensity and fluorescence quenching efficiency.

### Fluorescence assay of TET and reaction condition optimization of the developed biosensor

For TET detection, the Apt76-AgNCs was directly mixed with buffer of different TET concentrations and allowed to shake for 1 min. The mixes were incubated for 100 min at 37 °C and then measured the fluorescence intensity. Using the Apt76-AgNCs in the absence of TET as a control, TET concentration was proportional to the reduced fluorescence emission intensity at 625 nm (F_0_–F)/F_0_, where F_0_ and F represent the ensembled solution’s fluorescence emission with and without TET, respectively. PBS, Tris-HCl, Tris-hydrochloride, and Tris-citric acid Tris-citrate with a pH of 7.6 were used as reaction buffers to compare the fluorescence intensity and fluorescence quenching efficiency. The link between the luminescent intensity and the incubation time was then determined. Apt76-AgNCs and TET were mixed and tested every 10 min for 180 min.

### Fluorescence detection of TET in raw milk

The pure milk samples were obtained from a nearby supermarket. To eliminate interfering components from the milk, the process for pretreatment of milk was carried out in accordance with previous work [27], as follows. First, 3 mL of ultrapure water was used to dilute a milk sample (2 mL) spiked with TET. The aforementioned solution was then vortexed for 1–2 min with 1 mL of 10% trichloroacetic acid and 1 mL of trichloroacetic acid. Afterwards, the solution was ultrasonically treated for 15 min before being centrifuged at 12,000 rpm for 10 min to precipitate proteins and dissolve other organic debris in the matrix. Following that, the acquired supernatant was filtered into another centrifuge tube, spun again at 12,000 rpm for 10 min, pH 7.6 was corrected with 1 M NaOH, and the final resultant supernatant was utilized for fluorescence examination.

## Results and discussion

### Principle of the label-free biosensor based on AgNCs

**Scheme 1:**
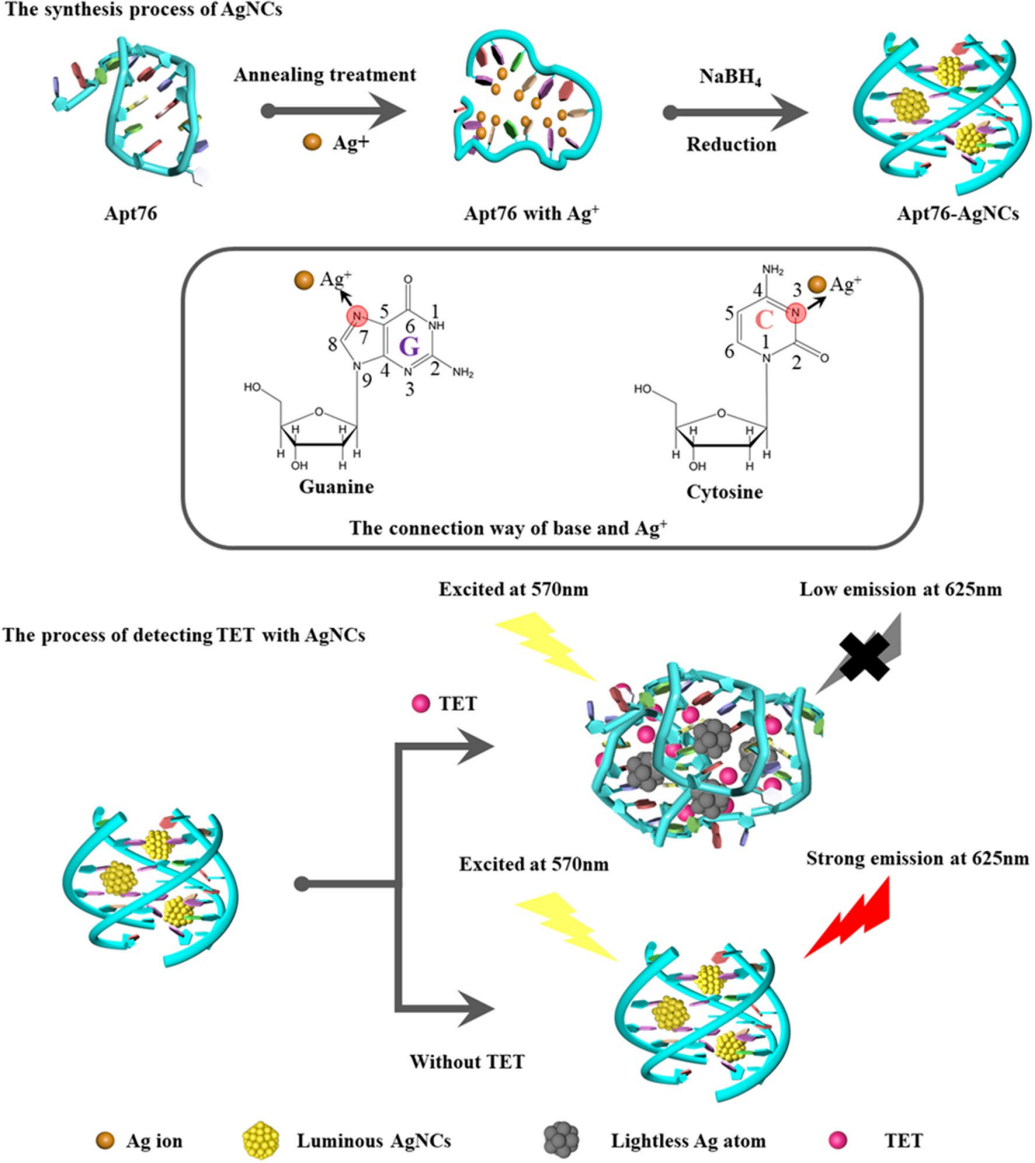
The principle of the label-free biosensor based on AgNCs for the detection of TET

The capacity of Apt76 to produce AgNCs underpins the proven label-free aptamer biosensor. As shown in Scheme 1, the established aptamer biosensor is using the ability of Apt76 to manufacture AgNCs, target-induced Apt76 conformation transition, and optical properties of AgNCs varied with the Apt76 conformation. The reason why DNA could be used as a template for AgNCs is that silver ions can combine with the N3 position of the cytosine bases and the N7 and O6 positions of the guanine bases are also preferred binding sites for silver ions [28]. Moreover, the DNA conformation is strongly influenced by sequence composition and affects the growth of AgNCs [29]. The content of G and C bases in Apt76 is as high as 68.4%, which provides a foundation for the synthesis of AgNCs. In addition, it has been reported that Apt76 in buffer can form a G-quadruplex structure [30], which is a suitable matrix for preventing AgNCs aggregation [21]. In this study, AgNCs synthesized with Apt76 as a template exhibited high fluorescence emission at 625 nm under stimulation at 570 nm. When target molecules (TET) were present, the shape of the aptamer specific for TET changed from an antiparallel G-quadruplex to a hairpin structure. TET is quantified based on the observed decrease in fluorescence emission at 625 nm produced by a change in Apt76 conformation upon specific binding to TET, which is followed by an increase in the size of the encapsulated AgNCs.

### Molecular docking results of Apt76 and TET

**Fig. 1:**
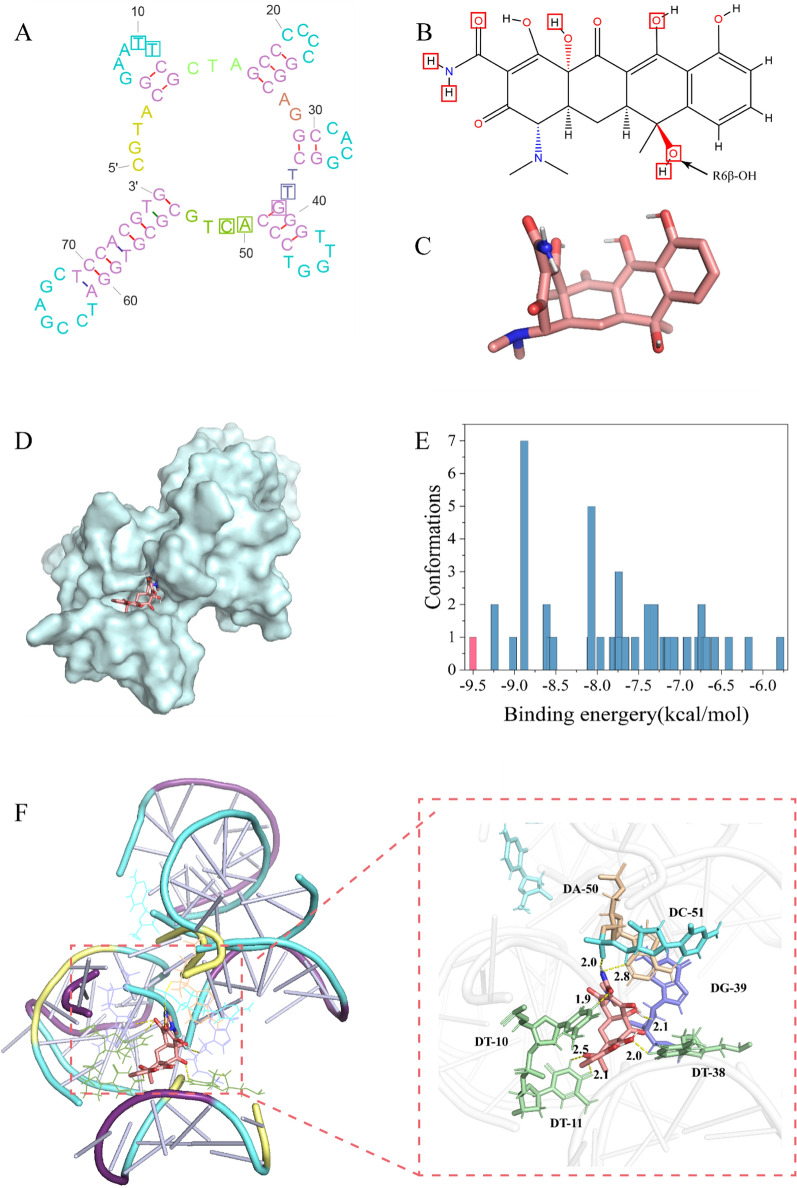
**A** Secondary structure of Apt76. **B** Chemical structure of TET. **C** The highest possible conformation of TET. **D** Molecular docking of TET with the parent aptamer. **E** Cluster analysis of the docking results of TET and the parent aptamer. **F** Hydrogen bonds between Apt76 and TET

Exploring the Apt76 recognition process requires revealing the exact binding sites of Apt76 with TET. AutoDock Tools was used to perform a molecular docking investigation of Apt76 and TET. As seen in Fig. 1E, the 50 Apt76 conformations with varying binding energies were simulated and distributed as 33 clusters, and the conformation signed with red column had the lowest binding energy and the best potential of functioning as the real conformation. The TET conformation corresponding to the red column is shown in Fig. 1C. Hydrogen bonding, shape matching, aromatic ring stacking, electrostatic adsorption and van der Waals contacts are all used in the identification of nucleic acid aptamers and target molecules, with hydrogen bonding being the most essential [31]. The global perspectives depicted in Fig. 1D illustrate that Apt76 folds to form ‘pocket’ sites to encapsulate TET. As shown in Fig. 1F, Apt76 resembles a complex s-shaped structure with the ligand-binding site in the depression of neck-loop stacking. Main contact occurs at thymine T11, which forms two hydrogen bonds with the 6β-OH group of tetracycline. The β-hydroxyketone moiety of TET is deeply buried in Apt76 and forms three hydrogen bonds with T10, C51 and A50. In addition, T38 and G3 also formed two hydrogen bonds with TET. The positions of hydrogen bonding on Apt76 and TET are marked in Fig. 1A, B, respectively. According to the results of the foregoing investigation, the quantity of binding free energy and the number of hydrogen bonds demonstrated Apt76’s significant binding capacity to TET.

### Optimization of Apt76-AgNCs synthesis conditions

**Fig. 2:**
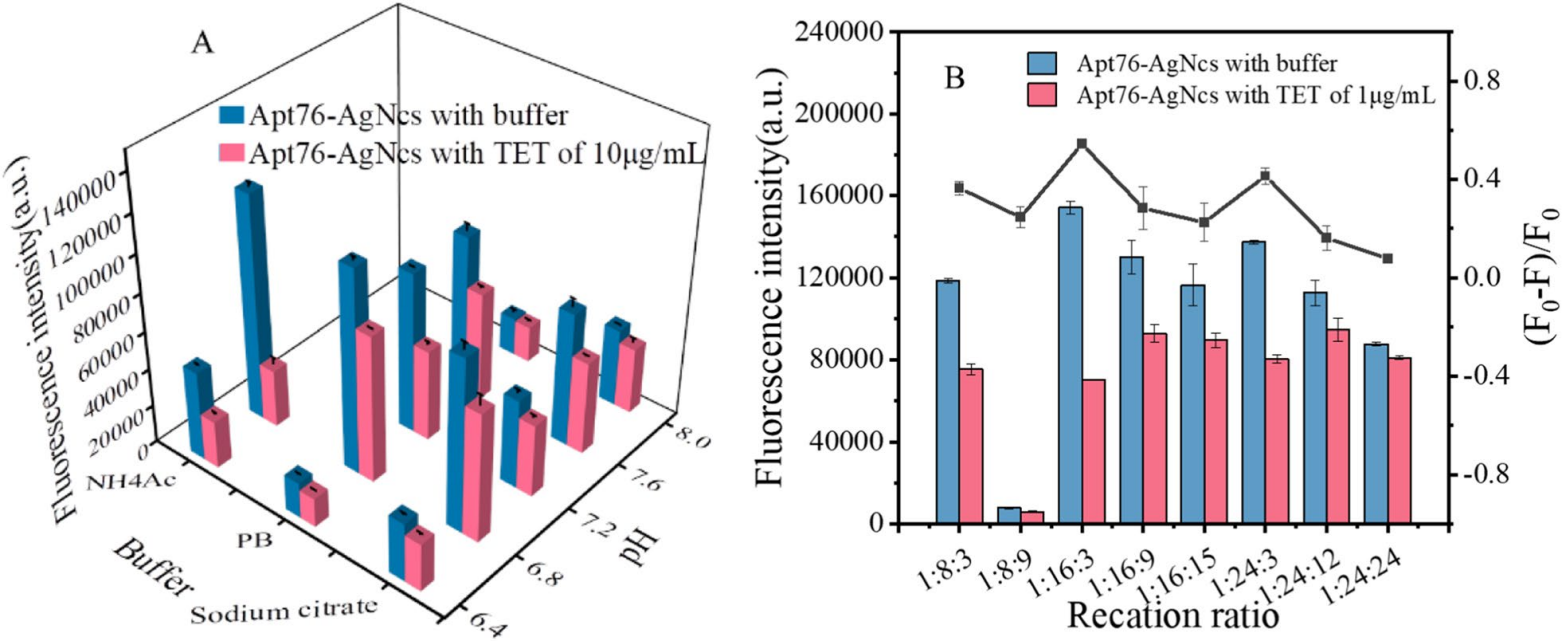
**A** Impact of the buffer and pH on the fluorescence intensity of DNA-AgNCs. **B** Impact of Apt76:Ag^+^:NaBH4 molar ratio on the fluorescence intensity of DNA-AgNCs and fluorescence quenching efficiency with TET.

The fluorescent property of DNA-AgNCs is the source of the biosensor signal, and the highest fluorescence intensity corresponds to the best detection sensitivity, as a result, various critical variables for the creation of Apt76-AgNCs must be optimized, including the synthesis buffers with different pH values and Apt76/Ag^+^/NaBH_4_ molar ratios. Fluorescence intensity and fluorescence quenching efficiency were achieved as quality indicators for AgNCs. First, the synthesis buffer for the synthesis of AgNCs was optimized. NH_4_Ac, PB and sodium citrate, which are commonly used to prepare AgNCs, were selected as synthesis buffers for comparison. Figure 2A shows the fluorescence intensity at 625 nm of AgNCs produced with various buffers under 570 nm excitation and fluorescence quenching efficiency in the presence of 10 µg/mL TET. It can be seen from Fig. 2A that under strong acidic and alkaline environments, it is not conducive to the synthesis of AgNCs. This is because N3 on cytosine more easily binds to Ag^+^ near neutral pH [32]. Apt76-AgNCs prepared in all three buffers have the highest fluorescence emission intensity at pH = 6.8, but only in NH_4_Ac buffer have the highest fluorescence quenching efficiency when reacting with TET. This outcome might be attributable to the fact that Na^+^ in PB and sodium citrate stabilize the G-quadruplex of Apt76 more strongly than NH4^+^ in NH4Ac, and when reacting with TET, the stable structure is harder to change. As a result, the decrease in fluorescence intensity is reduced [33]. Figure 2B depicts the influence of varied DNA to Ag^+^ and NaBH_4_ ratios on the fluorescence intensity of AgNCs. With a molar ratio of Apt76:Ag^+^:NaBH_4_ of 1:12:3, Apt76-AgNCs showed the maximum fluorescence intensity as well as the greatest decrease in fluorescence signal when TET is present. The results indicate that under the same molar ratio of Apt76 and Ag^+^, the reducing agent concentration is not proportional to the fluorescence signal, because an excess concentration of reducing agent causes too much Ag^+^ to be reduced to Ag atoms, which transforms AgNCs into AgPCs without strong emission at 625 nm. Furthermore, a larger molar ratio of Ag^+^ to DNA is not preferable [34].

### Characterization of Apt76-AgNCs

**Fig. 3:**
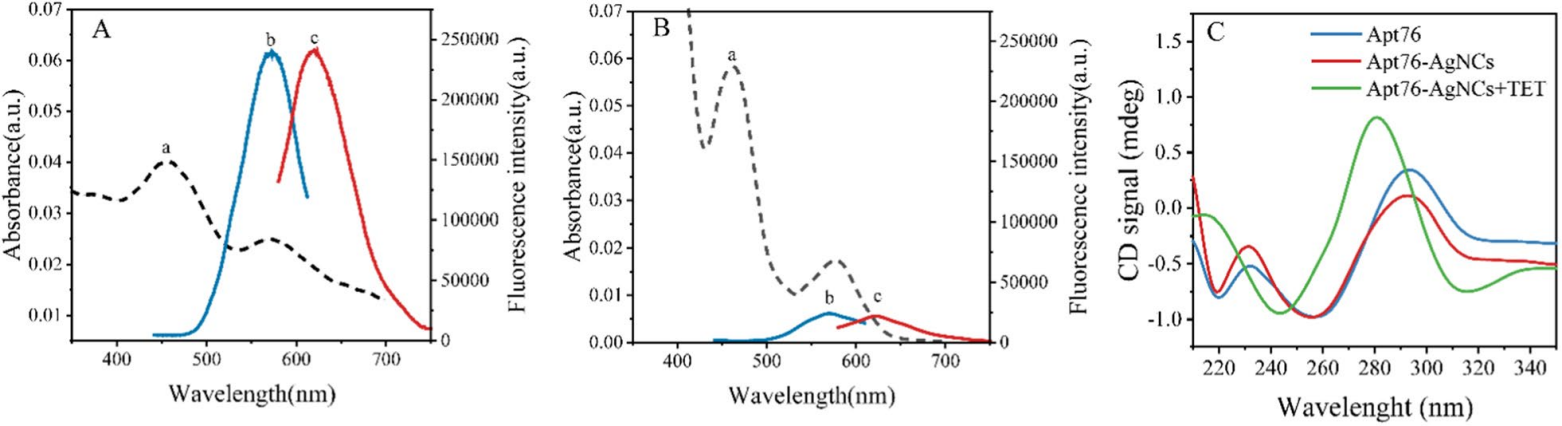
Optical characterization of Apt76-AgNCs without (**A**) and with 10 µg/mL TET (**B**). Absorption (curve a), excitation (curve b), and emission (curve c) spectra of the as-prepared Apt76-AgNCs. **C** Circular dichroism (CD) spectra of Apt76 alone, Apt76-AgNCs without and with 10 µg/mL TET.

**Fig. 4:**
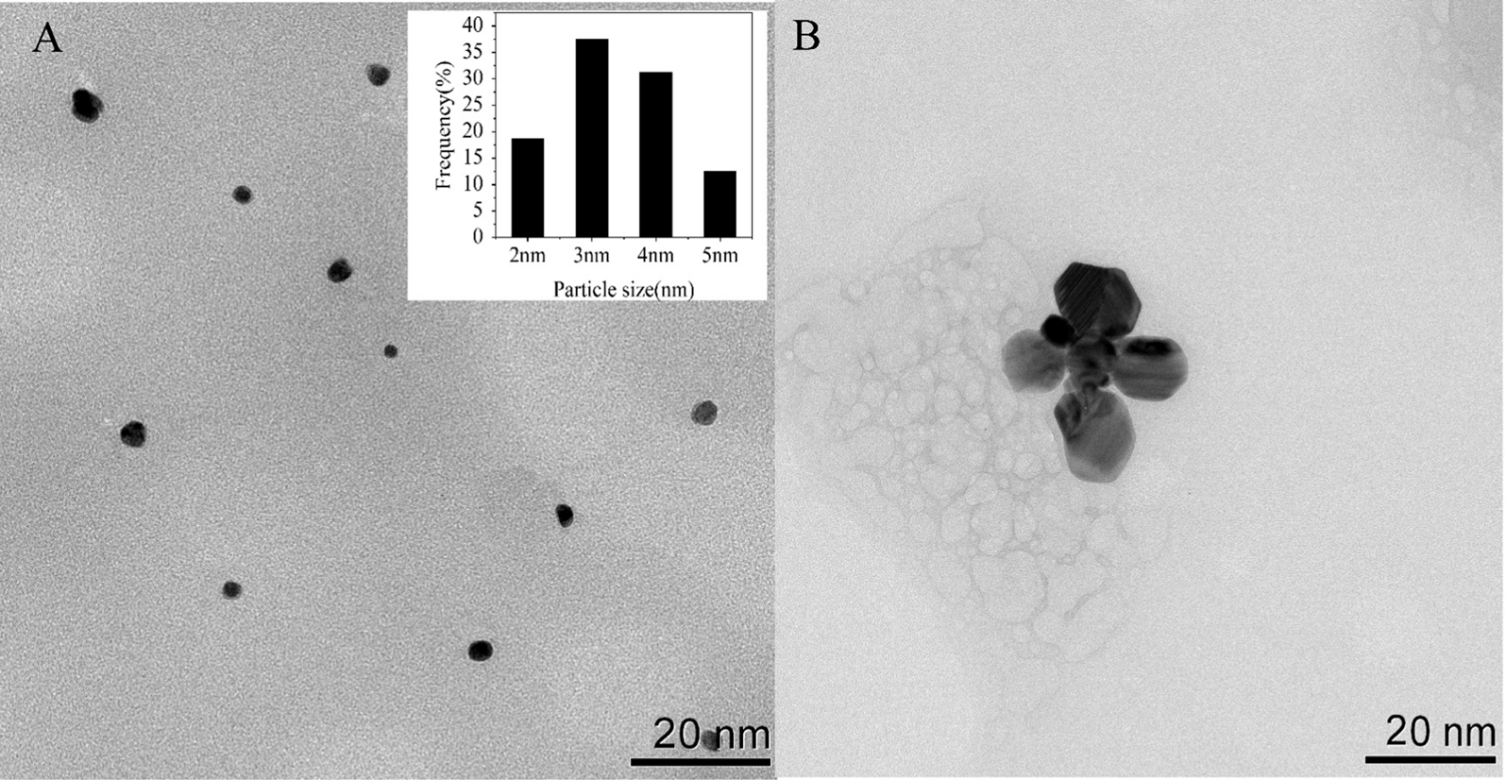
TEM image of Apt76-AgNCs without (**A**) and with 10 µg/mL TET (**B**)

To investigate the optical characteristics of Apt76-AgNCs without and with TET of 10 µg/mL, the UV–vis absorption and fluorescence spectra were studied. There are two peaks at 450 and 570 nm in both UV–vis absorption spectra of Apt76-AgNCs alone (Fig. 3A, curve a) and with 10 µg/mL TET (Fig. 3B, curve a) possess, as illustrated in Fig. 3. The peaks at 450 and 570 nm correspond to a typical absorption band of Ag nanoparticles [34] and AgNCs [35], respectively. With 10 µg/mL TET present, the fluorescence intensity at 625 nm of Apt76-AgNCs is weaker than Apt76-AgNCs alone, when excited at 570 nm (Fig. 3A, B, curve b and c), this is due to the fact that Apt76 folds into a hairpin form in the presence of TET, changing the size of AgNCs and causing fluorescence quenching. The Apt76-AgNCs were characterized with Transmission electron microscopy (TEM). Apt76-AgNCs without TET have a rather homogeneous distribution, as illustrated in Fig. 4, and the particle size is mostly 3 nm. However, we can see intuitively and plainly that the presence of TET causes AgNCs aggregation and size expansion, which explains why fluorescence characteristics of Apt76-AgNCs fluctuate with and without TET.

### Verification of the Apt76 conformation transition

According to our study, the aptamer biosensor responded sensitively with the transition of AgNCs to AgNPs, and the conformation transition of Apt76 played a role when TET was present. To confirm the principle of the biosensor, further verification experiments were carried out. From the circular dichroism (Fig. 3C), the original Apt76 showed a negative peak at around 260 nm and a positive peak at approximately 290 nm, validating the properties of the aptamer’s G-quadruplex structure [36]. After the synthesis of AgNCs based on Apt76, the peak value did not shift, but the CD signal reduced, which might be attributable to the existence of Ag^+^ and Ag atoms affecting Apt76 stability to a certain extent [37]. After reacting with TET, the negative peak of the CD spectrum of DNA-AgNCs shifted to approximately 245 nm, and the positive peak moved to approximately 280 nm, that is a typical CD spectrum feature of B- type DNA [38], marking the transition from the G-quadruplex to the hairpin structure transition [19].

### Optimization of the experimental conditions of the developed biosensor

**Fig. 5:**
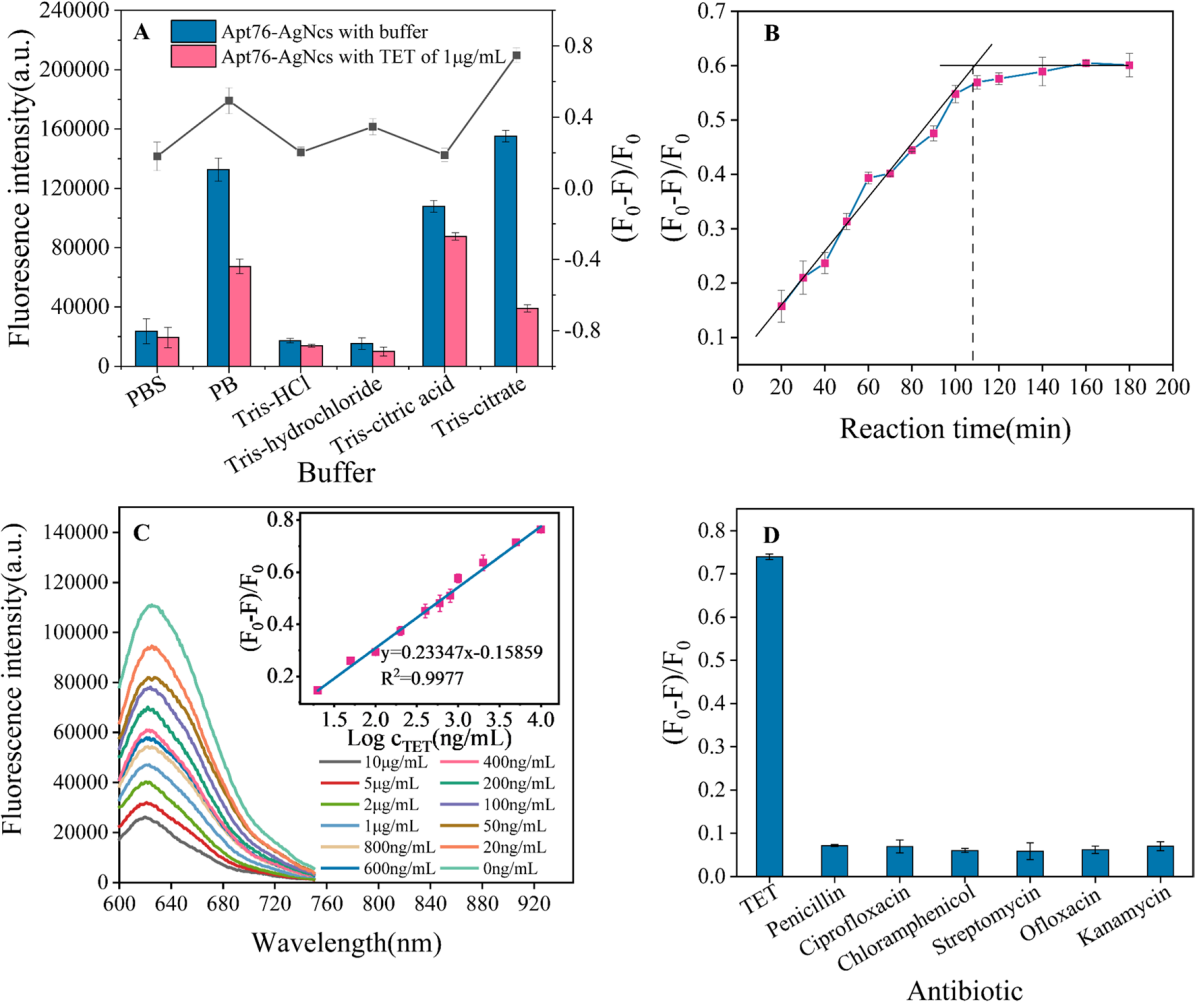
**A** Impact of buffer on the fluorescence intensity of Apt76-AgNCs and fluorescence quenching efficiency with TET. **B** Impact of the reaction time on the fluorescence quenching efficiency without TET. **C** Detection of TET using the developed biosensor. Fluorescence spectra with varied TET doses, as well as the biosensor’s response and analytical curves. **D** Selectivity of the sensor toward TET against other antibiotics (10 µg/mL). Three experiments resulted in error bars

The ability of Apt76-AgNCs to capture TET is also a key factor in determining the sensitivity of the designed biosensor. The decaying fluorescence signals can be detected more sensitively by assuring background fluorescence and boosting the reaction efficiency of Apt76 and TET as much as feasible. Therefore, it is necessary to optimize the reaction buffer. Figure 5A shows the fluorescence values of Apt76-AgNCs with and without TET and the fluorescence quenching efficiency of TET for different reaction buffers. It was reported that the optimal pH for the reaction between Apt76 and tetracycline was 7.6, so the pH of the buffers to be used was controlled at 7.6 [26]. The Tris-citrate buffer was prepared in strict accordance with the ion concentration of Apt76 screening buffer reported (5 mM K^+^,1 mM Ca^2+^,100 mM Na^+^,0.2 mM Mg^2+^,20 mM Tris), which not only guaranteed the fluorescence intensity of AgNCs but also ensured the binding ability of Apt76 to TET [26]. The buffer containing Cl^-^ (PBS, Tris-HCl and Tris-hydrochloride) was found to have very low fluorescence emission because Cl^-^ could form AgCl with Ag^+^, which indirectly quenched the fluorescence of AgNCs. In the buffer solution without metal ions (Tris-citric acid), although it still possessed a high fluorescence emission, it did not have a good fluorescence quenching efficiency, which indicated that Apt76 needed metal ions to maintain the pocket shape for capturing TET [39]. In PB buffer, the fluorescence quenching efficiency was lower than that of Tris-citrate, possibly due to the lack of divalent metal cations. The link between the signal and reaction time was studied by adding just Apt76-AgNCs and TET to the optimal buffer to identify the right time to develop this biosensor. To eliminate interference produced by taking constant measurements in a brief interval, the solution was examined every 10 min. During the first hundred minutes, the fluorescence intensity rapidly rose, as seen in Fig. 5B, and then leveled off. As a result, the reaction duration in this study was set at 100 min. Under the optimal conditions, the Apt76-AgNCs were prepared, and the fluorescence spectra with different concentrations of TET in buffer were measured. As seen in Fig. 5C, the absorption peaks at 625 nm diminished as the TET concentration rose. As a result, in the concentration range of 20 ng/mL–10 µg/mL was shown to have a strong linear connection with the signal intensity (R^2^ = 0.9977). The value of LOD was 11.46 ng/mL and equated to a concentration three standard deviations above the blank. Furthermore, the suggested approach is equivalent to those of the published biosensors for TET in Table 1, due to its low LOD, easy operation, and quick detection time, as well as the absence of chemical labeling.

**Table 1:** Comparison of aptasensors for TET detection

Methods	Linear range (ng/mL)	LOD (ng/mL)	Transducer type	Time needed	References
Fluorescent aptasensor based on SYBR Green I	5000–25,000	100	Fluorescence	20 min	[5]
Colorimetric aptasensor based on AuNPs	0.675–22.5	0.5985	Colorimetry	3 h 55 min	[40]
Aptasensor based on liquid crystal	0.0011–1.125	0.0011	Imaging	> 11 h	[41]
Fluorescent aptasensor based on thiazole orange	50-100000	29	Fluorescence	20 min	[42]
Instrument-free visual aptasensor based on caged G-quadruplex and DNAzyme	0.0023-225	0.00045	Colorimetry	–	[43]
Fluorescent aptasensor based on graphene quantum dots and palladium nanoparticles	50–100	45	Fluorescent	8 h	[16]
SPR aptasensor based on oriented aptamer	0.01–1000	0.0978	SPR signal	1 h	[44]
Fluorescent aptasensor based on Apt76-AgNCs	20–10,000	11.46	Fluorescence	100 min	This work

### Sensitivity and selectivity of TET detection

Specificity is an essential assessment parameter for biosensor sensing performance. Several antibiotics, including penicillin, ciprofloxacin, chloramphenicol (CAP), streptomycin, ofloxacin and aminoglycosides (KANA), were tested under optimal conditions with 10 µg/mL. Only TET may produce considerable variation in the biosensor signal, as seen in Fig. 5D, and the related fluorescence quenching efficiency is near to 75%. Other antibiotics, on the other hand, cannot induce a considerable decline in biosensor signal. These findings show that the suggested fluorescent approach has acceptable specificity for TET. The remarkable selectivity is due to the Apt76’s unusual three-dimensional structure, which generates high affinity interactions with TET and can distinguish the TET and other antibiotics based on minor structural differences.

### Analysis of TET in raw milk with the developed biosensor

The proposed sensor was used to test TET in milk to evaluate the dependability and applicability of the AgNCs biosensor in real samples. The milk was pretreated in accordance with the technique outlined in previous description. TET (20, 50, 100, 200, and 500 ng/mL) was added to the final supernatant and then the biosensor was used to evaluate the spiked samples. As shown in Table 2, the result indicates that the recoveries of the spiked samples varied from 97.7 to 114.6%, and the relative standard deviations (RSDs) are in the range of 2.8–8.5%. To further validate the accuracy of the proposed method, the TET content of the same samples was measured by HPLC method. The results obtained by the two methods are consistent in Table 2. As a result, the suggested biosensor offers a wide range of applications in milk detection, with excellent precision and accuracy.

**Table 2:** Recoveries of TET from milk samples (n = 5)

The proposed method					HPLC method	
Sample	Added (ng/mL)	Found (ng/mL)	RSD (%)	Recovery (%)	Found (ng/mL)	Recovery (%)
1	0	1.15	0.9	–	–	–
2	20	19.5	8.5	97.7	21.1	105.3
3	50	52.7	6.5	105.3	49.2	98.4
4	100	114.6	4.5	114.6	108.0	108.0
5	200	223.3	2.9	111.6	208.8	104.4
6	500	493.7	2.8	98.8	490.8	98.2

## Conclusion

We have created a simple, rapid and label-free fluorescent biosensor for TET detection. A novel AgNCs synthesize by Apt76 as both molecular recognition elements and signal transduction elements exhibits highly specific binding ability with TET and shows high fluorescence emission near 625 nm. The established biosensor is based on the fluorescence reduction caused by conformation changes in Apt76-AgNCs due to their effective interaction with TET. The fluorescence signal response is in a strong linear connection with the concentration of TET from 20 ng/mL to 10 µg/mL. Moreover, the biosensor exhibits excellent applicability in assessing TET in practical analysis, with recoveries of 97.7–114.6% and high specificity for TET detection. The perfect combination of aptamers and AgNCs effectively integrates the molecular recognition part and signal response part of the sensor without the need for chemical modification, which simplifies the fabrication process of the sensor, reduces the cost and enables high-throughput detection. It can be extended to other aptamers with specific binding ability to small molecules as templates for AgNCs synthesis to build faster label-free sensors. In addition, the emissive wavelength of DNA-AgNCs can be changed across a large range by using different aptamer sequences, which makes it possible to detect multiple substances simultaneously. It is expected that this novel strategy will provide new ideas for other small molecules to screen new specific binding aptamers in the future. This novel strategy is likely to inspire additional small molecules to screen for new specialized binding aptamers in the future.

## Data Availability

The data are all available upon request.

## References

[1] Granados-Chinchilla F, Rodríguez C (2017). Tetracyclines in food and feedingstuffs: from regulation to analytical methods, bacterial resistance, and environmental and health implications. J Anal Methods Chem.

[2] Muriuki FK, Ogara WO, Njeruh FM, Mitema ES (2001). Tetracycline residue levels in cattle meat from Nairobi salughter house in Kenya. J Vet Sci.

[3] De Ruyck H, De Ridder H, Van Renterghem R, Van Wambeke F (1999). Validation of HPLC method of analysis of tetracycline residues in eggs and broiler meat and its application to a feeding trial. Food Addit Contam.

[4] Vragović N, Bažulić D, Njari B (2011). Risk assessment of streptomycin and tetracycline residues in meat and milk on croatian market. Food Chem Toxicol.

[5] Viñas P, Balsalobre N, López-Erroz C, Hernández-Córdoba M (2004). Liquid chromatography with ultraviolet absorbance detection for the analysis of tetracycline residues in honey. J Chromatogr A.

[6] Miller CS, McGartty GJ (2009). Tetracycline-induced renal failure after dental treatment. J Am Dent Assoc.

[7] Pastor-Navarro N, Morais S, Maquieira Á, Puchades R (2007). Synthesis of haptens and development of a sensitive immunoassay for tetracycline residues. Application to honey samples. Anal Chim Acta.

[8] Fritz JW, Zuo Y (2007). Simultaneous determination of tetracycline, oxytetracycline, and 4-epitetracycline in milk by high-performance liquid chromatography. Food Chem.

[9] Nozal L, Arce L, Simonet BM, Ríos A, Valcárcel M (2004). Rapid determination of trace levels of tetracyclines in surface water using a continuous flow manifold coupled to a capillary electrophoresis system. Anal Chim Acta.

[10] Martins MT, Barreto F, Hoff RB, Jank L, Arsand JB, Feijó TC (2015). Determination of quinolones and fluoroquinolones, tetracyclines and sulfonamides in bovine, swine and poultry liver using LC-MS/MS.. Food Addit Contam Part A Chem.

[11] Zhou P, Lu Y, Zhu J, Hong J, Li B, Zhou J (2004). Nanocolloidal gold-based immunoassay for the detection of the N-methylcarbamate pesticide carbofuran. J Agric Food Chem.

[12] Chen Y, Kong D, Liu L, Song S, Kuang H, Xu C (2016). Development of an ELISA and immunochromatographic assay for tetracycline, oxytetracycline, and chlortetracycline residues in milk and honey based on the class-specific monoclonal antibody. Food Anal Methods.

[13] Bai F, Bu T, Zhang M, Tian Y, Sun X, Jia P (2020). Rhombic-like Al nanosupporter-based fluorescent immunochromatographic assay for the sensitive detection of tetracycline. Sensors Actuators B Chem.

[14] Xie N, Wang H, Quan K, Feng F, Huang J, Wang K (2020). Self-assembled DNA-based geometric polyhedrons: construction and applications. Trends Anal Chem..

[15] Ouyang Q, Liu Y, Chen Q, Guo Z, Zhao J, Li H (2017). Rapid and specific sensing of tetracycline in food using a novel upconversion aptasensor. Food Control.

[16] He H, Xie C, Yao L, Ning G, Wang Y (2021). A sensitive fluorescent assay for tetracycline detection based on triple-helix aptamer probe and cyclodextrin supramolecular inclusion. J Fluoresc.

[17] Zhang L, Wang J, Deng J, Wang S (2020). A novel fluorescent “turn-on” aptasensor based on nitrogen-doped graphene quantum dots and hexagonal cobalt oxyhydroxide nanoflakes to detect tetracycline. Anal Bioanal Chem.

[18] Ahmed SR, Kumar S, Ortega GA, Srinivasan S, Rajabzadeh AR (2021). Target specific aptamer-induced self-assembly of fluorescent graphene quantum dots on palladium nanoparticles for sensitive detection of tetracycline in raw milk. Food Chem.

[19] Yang C, Bie J, Zhang X, Yan C, Li H, Zhang M (2018). A label-free aptasensor for the detection of tetracycline based on the luminescence of SYBR Green I. Spectrochim Acta Part A Mol Biomol Spectrosc..

[20] Han D, Wei C (2018). A molecular beacon based on DNA-templated silver nanoclusters for the highly sensitive and selective multiplexed detection of virulence genes. Talanta.

[21] Tao G, Chen Y, Lin R, Zhou J, Pei X, Liu F (2018). How G-quadruplex topology and loop sequences affect optical properties of DNA-templated silver nanoclusters. Nano Res.

[22] Sengupta B, Springer K, Buckman JG, Story SP, Abe OH, Hasan ZW (2009). DNA templates for fluorescent silver clusters and i-motif folding. J Phys Chem C.

[23] Yan X, Sun J, Zhao XE, Wang R, Wang X, Zuo YN (2018). Molecular beacon-templated silver nanoclusters as a fluorescent probe for determination of bleomycin via DNA scission. Microchim Acta.

[24] Jiang Y, Ma X, Shao X, Wang M, Jiang Y, Miao P (2019). Chameleon silver nanoclusters for ratiometric sensing of miRNA. Sensors Actuators B Chem.

[25] Jiang Y, Tang Y, Miao P (2019). Polydopamine nanosphere@silver nanoclusters for fluorescence detection of multiplex tumor markers. Nanoscale R Soc Chem.

[26] Niazi JH, Lee SJ, Gu MB (2008). Single-stranded DNA aptamers specific for antibiotics tetracyclines. Bioorg Med Chem.

[27] Ping H, Zhang M, Li H, Li S, Chen Q, Sun C (2012). Visual detection of melamine in raw milk by label-free silver nanoparticles. Food Control.

[28] Liu J (2014). DNA-stabilized, fluorescent, metal nanoclusters for biosensor development. Trends Anal Chem.

[29] Ritchie CM, Johnsen KR, Kiser JR, Antoku Y, Dickson RM, Petty JT (2007). Ag nanocluster formation using a cytosine oligonucleotide template. J Phys Chem C.

[30] Dai Y, Zhang Y, Liao W, Wang W, Wu L (2020). G-quadruplex specific thioflavin T-based label-free fluorescence aptasensor for rapid detection of tetracycline. Spectrochim Acta Part A Mol Biomol Spectrosc.

[31] Hermann T, Patel DJ (2000). Adaptive recognition by nucleic acid aptamers. Science (80-).

[32] Kennedy TAC, MacLean JL, Liu J (2012). Blue emitting gold nanoclusters templated by poly-cytosine DNA at low pH and poly-adenine DNA at neutral pH. Chem Commun.

[33] Smargiasso N, Hsia W, Colson P, Baker ES, Bowers MT, Pauw E, De (2008). G-Quadruplex DNA assemblies: loop length, Cation Identity, and multimer formation †. J Am Chem Soc.

[34] Fornell C, Larcker DF (1981). Solution structural studies of the ag(I)-DNA complex. Nucleic Acids Res.

[35] Zhang B, Wei C (2018). Highly sensitive and selective detection of Pb2 + using a turn-on fluorescent aptamer DNA silver nanoclusters sensor. Talanta.

[36] Kypr J, Kejnovská I, Renčiuk D, Vorlíčková M (2009). Circular dichroism and conformational polymorphism of DNA. Nucleic Acids Res.

[37] Fu Y, Zhang J, Chen X, Huang T, Duan X, Li W (2011). Silver nanomaterials regulated by structural competition of G-/C-rich oligonucleotides. J Phys Chem C.

[38] Birader K, Kumar P, Tammineni Y, Barla JA, Reddy S, Suman P (2021). Colorimetric aptasensor for on-site detection of oxytetracycline antibiotic in milk. Food Chem.

[39] Wang S, Yong W, Liu J, Zhang L, Chen Q, Dong Y (2014). Development of an indirect competitive assay-based aptasensor for highly sensitive detection of tetracycline residue in honey. Biosens Bioelectron.

[40] Zhou L, Li DJ, Gai L, Wang JP, Li Y, Bin, (2012). Electrochemical aptasensor for the detection of tetracycline with multi-walled carbon nanotubes amplification. Sensors Actuators B Chem..

[41] Wang S, Gao S, Sun S, Yang Y, Zhang Y, Liu J (2016). A molecular recognition assisted colorimetric aptasensor for tetracycline. RSC Adv R Soc Chem.

[42] Ramezani M, Mohammad Danesh N, Lavaee P, Abnous K, Mohammad Taghdisi S (2015). A novel colorimetric triple-helix molecular switch aptasensor for ultrasensitive detection of tetracycline. Biosens Bioelectron.

[43] Chen J, Chen S, Li F (2017). Instrument-free visual detection of tetracycline on an autocatalytic DNA machine using a caged G-quadruplex as the signal reporter. Chem Commun.

[44] Noghabi HS, Abnous K, Taghdisi SM, Chamsaz M (2021). A novel fluorescent aptasensor for sensitive detection of oxytetracycline based on gold nanoparticles and OTC-Eu31 complex using two different methods for modification of gold nanoparticles. Aust J Chem.

